# Study on the Mechanism and Modification of Carbon-Based Materials for Pollutant Treatment

**DOI:** 10.3390/ma18235345

**Published:** 2025-11-27

**Authors:** Lingyi Meng, Zitong Shao, Wenqi Li, Jianxiong Wang, Changqing Hu, Guangqing Yang, Yan Shi

**Affiliations:** 1 College of Metallurgy and Energy, North China University of Science and Technology, Tangshan 063000, China; menglingyi@ncst.edu.cn (L.M.); wjx1104496681@163.com (J.W.); hiq-73@163.com (C.H.); yangguangqing@ncst.edu.cn (G.Y.); 2 Laboratory of Modern Metallurgy Technology, North China University of Science and Technology, Tangshan 063000, China; 3 College of Architecture and Engineering, North China University of Science and Technology, Tangshan 063000, China; m15232167989@163.com (Z.S.); livinciwenqi@163.com (W.L.)

**Keywords:** carbon-based materials, pollutant treatment, loss mechanism, modification technology, adsorption performance, catalytic activity, environmental remediation

## Abstract

**Highlights:**

**What are the main findings?**

- The core mechanisms by which carbon-based materials treat water, atmospheric, and soil pollutants have been elucidated, including adsorption mechanisms (clearly distinguishing between physical and chemical adsorption) and physicochemical degradation mechanisms. Concurrently, the degradation characteristics of different carbon-based materials and their specific impacts on pollutant treatment efficacy have been analyzed.
- Integrating new findings in the field, targeted physicochemical modification strategies have been proposed to effectively overcome the limitations of existing carbon-based materials in pollutant treatment.

**What are the implications of the main findings?**

- Practical Application Value: The modified carbon-based materials developed through this modification strategy significantly enhance pollutant adsorption efficiency and improve material regeneration capacity while reducing industrial application costs. This achieves a balance between environmental protection requirements and practical production needs.
- Technical and Engineering Value: This work charts the course for carbon-based material technology development, outlines the prospects for green intelligent modification techniques, and proposes equipment optimization solutions aligned with industrial needs. It delivers engineered solutions for multi-media synergistic pollutant remediation, propelling the technology from laboratory research to practical industrial application.

**Abstract:**

The implementation of ultra-low emission standards in the steel industry imposes higher demands on flue gas purification. Carbon-based materials, leveraging their porous structure and surface activity, demonstrate high adsorption potential for treating heavy metal ions, sulfur- and nitrogen-containing compounds, and volatile organic pollutants. However, their application is constrained by a limited selective adsorption capacity. This paper systematically analyzes the mechanisms by which carbon-based materials treat water, air, and soil pollutants; investigates their physical and chemical degradation patterns; and summarizes practical physicochemical modification pathways. Research indicates that modification techniques can effectively overcome performance limitations of carbon-based materials, enhance pollutant adsorption efficiency, and provide new insights for the engineering application of multi-media pollution synergistic control and environmental remediation technologies.

## 1. Introduction

With the acceleration of global industrialization and urbanization, water and air pollution have become prominent problems that threaten the ecological environment and human health.

**Figure 1:**
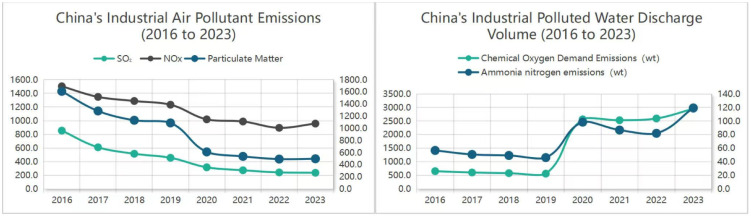
2016–2023 China’s industrial waste gas and industrial sewage discharge line chart.

According to the “China Ecological Environment Bulletin” [1] (Figure 1), the proportion of heavily polluted days in China decreased by 0.9% in 2024 compared to 2023. However, factors such as high-energy-consuming industrial structures and the rigid growth of energy consumption have pushed air pollution control into a more challenging phase. The difficulty of coordinated control of pollutants such as fluoride, SO_2_, and PM2.5 in industrial waste gas has increased, and the decline range has decreased year by year. At the same time, the traditional technologies for the treatment of heavy metals, organic solvents, and “permanent chemical substances” PFAS in lithium battery wastewater and chemical wastewater are also facing bottlenecks, and materials and technologies with better performance are urgently needed to break through the existing difficulties.

Among numerous adsorbent materials, carbon-based materials leverage their unique physical and chemical adsorption capabilities to effectively remove sulfur- and nitrogen-containing pollutants from the atmosphere, heavy metal ions from water bodies, and organic pollutants from soil [2]. As environmental standards continue to tighten, activated carbon technology demonstrates significant advantages in energy conservation and consumption reduction. Its hierarchical pore structure and enormous specific surface area facilitate efficient capture of diverse pollutants, while surface functional groups enable synergistic removal of multiple contaminants. Furthermore, carbon-based materials reduce pollutant mobility and biotoxicity through structural encapsulation and surface anchoring, promoting microbial degradation. This provides a practical and highly effective material foundation for addressing pollution across multiple environmental media. Although carbon-based materials have many advantages in the treatment of pollutants, they still have limitations. The following three aspects are described: (1) In the treatment of water pollution, the selective adsorption capacity of complex water pollutants is weak, which is susceptible to interference from coexisting ions and humus, and it is difficult to accurately target specific pollutants. (2) In the treatment of metallurgical industrial waste gas, high temperature, high humidity, and high concentration of pollutants will lead to the attenuation of catalytic activity of materials, and the synergistic mechanism of multi-pollutants is not clear. (3) In the treatment of soil pollutants, the large-scale preparation of biochar has high energy consumption and poor stability of raw materials, which may change the microbial community structure and have potential ecological risks. The mineralization efficiency of complex organic pollutants in solid waste is low, and easy to cause secondary pollution.

In addition to the aforementioned advantages, carbon-based materials also face issues of physical and chemical losses during practical use. Physical losses can easily cause damage to the pore structure, while chemical losses may lead to the deactivation of active sites. The combined effects of both will weaken the material’s overall adsorption and catalytic performance, thereby increasing operational costs.

Therefore, modification techniques for carbon-based materials have become a key approach to overcoming these limitations [3]. Physical modifications (e.g., high-temperature heat treatment and steam/CO_2_ activation) optimize pore structures and remove impurities, while chemical modifications (e.g., oxidation, reduction, and metal loading) introduce active functional groups. These approaches significantly enhance the materials’ selectivity, stability, and treatment efficiency toward target pollutants. Such modification techniques are crucial for advancing synergistic multi-pollutant remediation and achieving theoretical innovation and engineering translation in environmental remediation technologies.

Against this backdrop, this paper systematically reviews key process technologies and reaction mechanisms of carbon-based materials in pollutant treatment, summarizes recent research advances in this field, and focuses on analyzing material failure mechanisms and corresponding modification strategies. Building upon this foundation, it further explores critical issues in the treatment and modification of carbon-based materials and outlines future research directions. This clarifies the study’s objectives and scope, providing a theoretical reference for in-depth exploration in related fields.

## 2. Application of Carbon-Based Materials in Pollutant Treatment

### 2.1. Treatment of Water Environmental Pollutants

The pollution treatment of heavy metal ions, organic pollutants, and emerging pollutants (such as perfluorinated compounds, PFAS) in the water environment of industrial wastewater is becoming more and more serious. Different pollutant sources, components, and environmental impacts are different (as shown in Table 1). Traditional treatment technologies for these pollutants, such as chemical precipitation, are prone to secondary sludge pollution due to excessive pharmaceuticals. Membrane separation technology is limited by the attenuation of treatment efficiency and high maintenance costs caused by membrane fouling.

**Figure 2:**
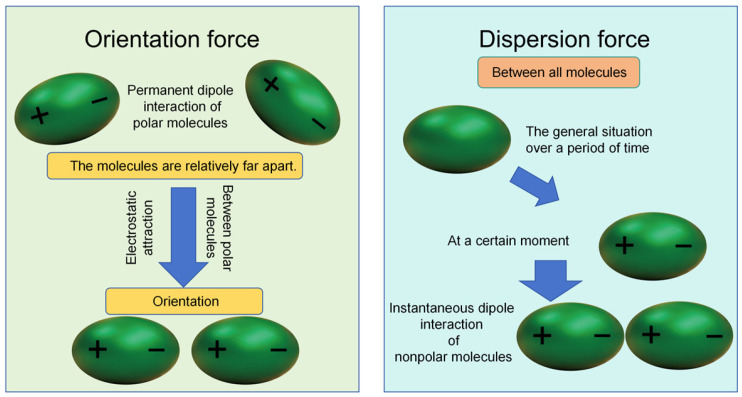
Schematic diagram of dispersion forces and orientation forces.

Activated carbon demonstrates significant advantages in adsorbing water pollutants due to its unique porous structure, surface chemistry, and high specific surface area, making it a crucial material for addressing water pollution issues. Physical adsorption: The ultra-large specific surface area of modified carbon-based materials (graphene-based activated carbon can reach up to 3505 m^2^/g) provides ample adsorption sites for pollutants [4] while matching pore sizes with the kinetic diameters of target pollutant molecules (e.g., the efficient capture of benzene by 1.997 nm pores) [5]. The core mechanism of activated carbon in treating heavy metal pollutants, organic pollutants, and nitrogen/phosphorus nutrient pollutants in aquatic environments remains consistent, relying on intermolecular forces and pore structure entrapment [6,7]. Wu et al. [8] successfully prepared high-performance modified activated carbon using biomass beet pulp as raw material, subjected to steam activation followed by reflux modification with 70% nitric acid solution. Characterization revealed a significant increase in oxygen-containing functional groups, with a specific surface area reaching 1322 m^2^/g. The material demonstrated strong adsorption capacity for both methylene blue and iodine. In Cd^2+^ adsorption experiments, under optimized conditions (25 °C, pH 8.5, 4 h adsorption), it achieved an 87% removal rate for 5 mg/L Cd^2+^ solutions. This material demonstrates promising application potential in the treatment of cadmium-containing wastewater. However, due to the different chemical forms and polarity of the three pollutants, the types of intermolecular forces that play a leading role in physical adsorption are different. Heavy metal pollutants and nitrogen and phosphorus nutrient pollutants usually exist in the form of ions, with strong polarity, and the main intermolecular force is the orientation force, while organic pollutants mainly exist in the form of molecules, and a few are polar molecules, so the main force of physical adsorption is the dispersion force. (As shown in Figure 2)

In terms of chemical adsorption:

Surface chemistry (e.g., reductive complexation of Cr (VI) with phenolic hydroxyl groups) [9,10].

Phenolic hydroxyl reduction:

(1) $${\mathrm{Cr}}_{2}O_{7}^{2-}+3C_{6}H_{4}\left(\mathrm{AC}\right)+8H^{+}\to 2{\mathrm{Cr}}^{3+}+7H_{2}O+3C_{6}H_{4}\mathrm{OH}$$

Hydroxyl reduction:

(2) $${\mathrm{Cr}}_{2}O_{7}^{2-}+3R\text{-}\mathrm{OH}\left(\mathrm{AC}\right)+2H^{+}\to 2{\mathrm{Cr}}^{3+}+7H_{2}O+3R\text{-}\mathrm{OH}\left(\mathrm{AC}\right)$$

The reaction can achieve selective adsorption of heavy metal ions (the adsorption capacity of Cr (VI) can still reach 90.63 mg/g after the regeneration of magnetic activated carbon by NaOH solution) and efficient enrichment of organic pollutants (for example, the adsorption capacities for Congo Red and methylene blue reached as high as 7265 mg/g and 2331 mg/g, respectively, while those for Rhodamine B and Methyl Orange ranged between 955 and 1327 mg/g) [11,12]. However, for nitrogen and phosphorus nutrients, the functional groups on the surface of activated carbon, such as carboxyl group (-COOH), hydroxyl group (-OH), and phenolic group, play a key role in chemical adsorption. The carboxyl group can react with ammonium ion (NH_4_^+^) to adsorb ammonia nitrogen in water, and the hydroxyl group can form a hydrogen bond with phosphate (PO_4_^3−^) to promote the adsorption of phosphorus [13,14].

**Figure 3:**
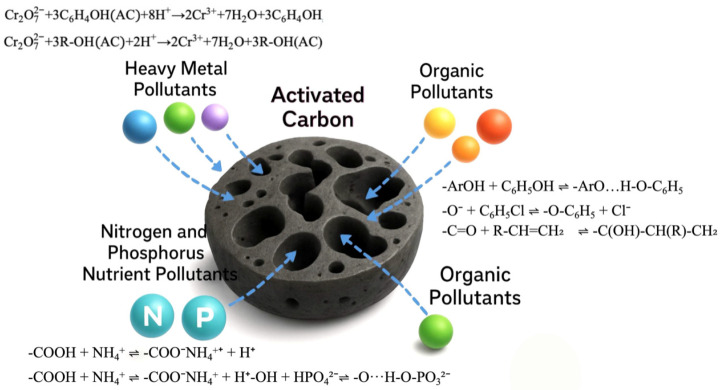
Activated carbon for heavy metals, organic matter, and nitrogen and phosphorus nutrient adsorption principle diagram.

The adsorption equation is as follows: (As shown in Figure 3)

Carboxyl and ammonium ions:

-COOH + NH_4_^+^ ⇌ -COO^−^NH_4_^+^ + H^+^ (3)

The hydrogen bonding interaction between hydroxyl and phosphate:

-OH + HPO_4_^2−^ ⇌ -OH-O-PO_3_^2−^ (4)

Therefore, activated carbon shows great application potential in the treatment of multiple pollutants in water pollution.

### 2.2. Air Pollutant Treatment

In the atmospheric environment, pollutants such as SO_2_, NO, and VOCs emitted from industrial waste gas and automobile exhaust seriously threaten air quality (As shown in Table 2).

**Figure 4:**
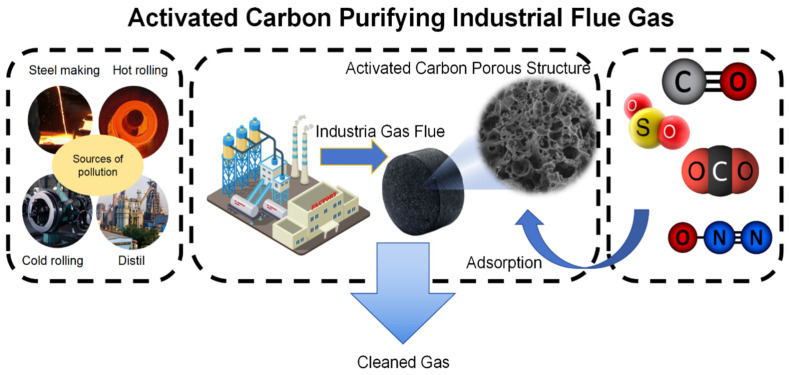
Principle diagram of activated carbon treatment of industrial waste gas.

Traditional technologies, such as wet desulfurization, can easily cause equipment corrosion, while the use of selective catalytic reduction (SCR) has the risk of catalyst deactivation and secondary pollution. However, activated carbon-based materials provide a new way for the treatment of air pollutants due to their unique structure and properties. The adsorption process of carbon-based materials for these three types of air pollutants can also be divided into physical adsorption and chemical adsorption (As shown in Figure 4).

In the process of physical adsorption, the adsorption of SO_2_ and NO is mainly dependent on the dipole–dipole interaction between molecules. The polarity of pollutant molecules and the weak polar sites on the surface of activated carbon attract each other [18]. At the same time, the microporous structure confines the small molecules SO_2_ and NO in the pores through the steric hindrance effect [19]. VOCs are mostly non-polar or weakly polar molecules, mainly adsorbed by the van der Waals force. The mesopores and micropores of activated carbon can be screened by the “molecular sieve effect” and intercept VOC molecules matching the pore size [20]. Han Xiao [21] team used kapok fiber as a raw material to prepare activated carbon fiber by the NaOH and ZnCl_2_ activation method. The specific surface area of activated carbon fiber was 1397 m^2^/g, and the microporous structure was dominant. At 30 °C, the adsorption capacity of toluene is as high as 479 mg/g, and the adsorption capacity of benzene is 350 mg/g, which significantly exceeds the threshold of 150 mg/g. The selective adsorption of VOCs with different molecular weights can be optimized by adjusting the pore structure.

In terms of chemical adsorption, industrial flue gas desulfurization is used as an example for SO_2_. While SO_2_ is physically adsorbed and enriched by activated carbon, functional groups such as phenolic hydroxyl groups and carbonyl groups on the surface can promote its oxidation. Part of SO_2_ is converted to SO_3_ and then reacts with water to generate H_2_SO_4_ to achieve pollutant fixation. The specific reaction process can be expressed as follows:

Physical adsorption of SO_2_:

(5) $${\mathrm{SO}}_{2}\left(g\right)\overset{\mathrm{AC}}{\to }{\mathrm{SO}}_{2}\left(\mathrm{ads}\right)$$

Surface catalytic oxidation:

(6) $$2{\mathrm{SO}}_{2}\left(\mathrm{ads}\right)+O_{2}\overset{\mathrm{Phenolic}\;\mathrm{hydroxyl},\;\mathrm{hydroxyl}}{\to }2{\mathrm{SO}}_{3}\left(\mathrm{ads}\right)$$

SO_3_ hydrates to form sulfuric acid:

(7) $${SO}_{3}+H_{2}O\to H_{2}{SO}_{4}$$

The overall reaction is as follows:

(8) $$2SO_{2}+O_{2}+2H_{2}O\overset{AC}{\to }2H_{2}{SO}_{4}$$

For NO_x_ (mainly NO and NO_2_), chemisorption depends on the reduction or oxidation sites on the surface of activated carbon: if there are reducing groups (such as phenolic hydroxyl groups) on the surface, NO_2_ can be reduced to NO_3_^−^ and combined with H^+^ to form -NO_3_^−^·H^+^ [22]; if modified by supported metal oxides, metal ions can form coordination bonds with NO, such as Cu^2+^ + NO → [Cu-NO]^2+^, achieving stable capture [23].

The chemical adsorption of VOCs is closely related to the molecular structure. VOCs containing double bonds or aromatic rings can undergo addition reactions with free radical sites on the surface of activated carbon [24]. VOCs containing hydroxyl (-OH) or carbonyl (C=O) can undergo condensation reaction with amino (-NH_2_) on the surface of activated carbon (-CHO + -NH_2_ → -CH=N- + H_2_O) [25]. Halogenated hydrocarbons may be combined with surface functional groups through a dehalogenation reaction to form a C-C covalent bond fixation. This strong chemical bond gives the material a high degree of chemical inertness and makes it not easy to react with acid/base substances in the atmosphere, thereby maintaining the structural integrity of the material [26]. Whether it is in the face of high-concentration acidic gases produced by industrial emissions or oxidizing free radicals in the urban atmosphere, carbon-based materials can avoid corrosion or decomposition by virtue of their stable chemical structure, ensure that their pore structure and active sites are not destroyed, and continue to play the role of pollutant adsorption and catalysis.

### 2.3. Soil Pollutant Treatment

In addition to the pollution in the water environment and the atmospheric environment, the pollution in the soil cannot be ignored. With the acceleration of the industrialization process, industrial wastewater, waste, and solid waste are directly discharged into the environment, resulting in soil pollution. Soil heavy metal pollution is widespread around mines and factories. Carbon-based materials also play an indispensable role in the treatment of soil pollutants.

Biochar has been widely used in soil carbon sequestration and heavy metal remediation. Yang [27] studied the effects of 1%, 2%, and 4% PBC, PKBC, and PNBC on contaminated soil in a manganese mine area for 100 days. PKBC and PNBC reduced cation exchange capacity, PNBC increased available phosphorus most significantly (90.99–431.45%), and PBC increased available potassium most significantly (18.95–92.2 times). A high proportion of PKBC and PNBC inhibited CO_2_ emissions and increased the content of organic carbon. PKBC and PNBC reduced acid-extractable Cd and increased oxidizable and residual Cd. Shi et al. [28] significantly improved the adsorption capacity of biochar to Cd^2+^ (up to 150 mg/g) through physical (ball milling and steam activation), chemical (acid/alkali modification and metal salt loading), and biological (microbial immobilization) modification strategies. The mechanism of action included surface complexation, electrostatic attraction, and ion exchange. Iron and manganese oxide-modified biochar can reduce the Cd content of rice by 19.47~33.02%. It can be seen that carbon-based materials play a role in adsorbing heavy metal pollutants and promoting biodegradation in soil environments polluted by heavy metals.

In summary, although biochar demonstrates significant potential in Cd^2+^ remediation, its standalone application remains limited in terms of adsorption capacity, stability, and environmental risks. To objectively evaluate its performance hierarchy and drive material optimization, it is imperative to systematically compare it with extensively studied materials such as clay minerals, zeolites, and their composites. The following section will present a critical analysis and comparison of these remediation materials through a tabular format, focusing on key performance parameters (As summarized in Table 3).

Organic pollutants in soil have hydrophobic properties, so the adsorption effect of carbon-based materials on pollutants is limited, which makes it difficult to have a significant remediation effect on contaminated soil. Biochar in carbon-based materials has the advantages of harmlessness and low cost for the remediation of organic-contaminated soil. Therefore, the modified biochar can still adsorb soil pollutants efficiently. Liu et al. [32] grafted β-cyclodextrin to Fe_3_O_4_@biochar to prepare magnetic solubilized biochar (CD@MBC), which was used to adsorb and repair benzo [a] pyrene (BaP) (PAHs model pollutant) contaminated soil. Studies have shown that CD@MBC has a large specific surface area, rich pore structure, and surface oxygen-containing functional groups, which are conducive to the adsorption and remediation of organic-contaminated soil. Under the conditions of CD@MBC addition amount of 1.00%, BC preparation temperature of 800 °C, solid–liquid ratio of 1:10, and adsorption temperature of 35 °C, the removal rate of BaP in contaminated soil by CD@MBC was up to 69.52%. It has a certain removal effect on soil organic pollution.

Carbon-based materials can efficiently adsorb soil pollutants. The reason is that the multi-level pore structure and surface functional groups are the key to giving them rapid adsorption equilibrium ability. Tang et al. [33] analyzed the adsorption form of pollutants, and the results showed that RHB and TC with obvious charge tended to achieve adsorption removal by single-layer adsorption, while DCP tended to multi-layer adsorption. In addition, in the single-layer adsorption system, the contribution of electrostatic interaction (charge amount) to adsorption is 5.13 times that of π-π interaction, while in the multi-layer adsorption system, the benzene ring structure of biochar (π-π interaction) is the main contribution site of adsorption.

The specific reaction formula is given in the article “Gold absorption method of biochar” [34] of the China Digital Science and Technology Museum.

Taking cation-π as an example:

C–π + 2H_2_O → C–π–H_3_O^+^ + OH–C–π–H_3_O^+^ + M → C–π–M + H_3_O^+^ (M represents heavy metal) (9)

At the same time, there is also ion exchange: there are a large number of acidic functional groups, such as hydroxyl and carboxyl groups, on the surface of biochar, which can provide H+ + for ion exchange with heavy metal ions.

The general reaction equation can be expressed as

2Surf–OH + M^2+^ → (Surf–O)2M + 2H^+^ (exchange with surface acidic functional groups, M represents heavy metals); (10)

2Surf–ONa + M^2+^ → (Surf–O)2M + 2Na^+^ (ion exchange with surface base, M represents heavy metal). (11)

The complexation reaction is also indispensable. The surface of biochar is rich in carboxyl groups, phosphoryl groups, hydroxyl groups, sulfate groups, amino groups, and amide groups. The hydrogen, nitrogen, oxygen, phosphorus, and sulfur in them can be used as coordination atoms to coordinate with heavy metal ions. The functional groups involved in surface complexation are mainly oxygen-containing functional groups, especially carboxyl groups and phenolic hydroxyl groups. The general formula of the reaction can be expressed as follows:

Surf–OH + M^2+^ + H_2_O → Surf–OM– + H_3_O^+^ (M represents heavy metals); (12)

Surf–COOH + M^2+^ + H_2_O → Surf–COOM– + H_3_O^+^ (M represents heavy metal) (13)

In summary, in the treatment of soil heavy metal and organic pollution caused by industrialization, carbon-based materials have become one of the core remediation materials with unique advantages. Biochar and modified products (PKBC, PNBC, etc.) can synergistically achieve soil nutrient regulation and heavy metal stability. Modified biochar can also break through the hydrophobic limitation of organic pollutants. Its multi-level pore structure and surface functional groups ensure the adsorption efficiency through multiple mechanisms. It provides an efficient and feasible technical path and theoretical support for soil pollution remediation, and has far-reaching significance for soil ecological protection and sustainable utilization.

## 3. Loss Mechanism of Carbon-Based Materials in the Pollutant Treatment Process

### 3.1. Carbon-Based Material Loss Overview

**Figure 5:**
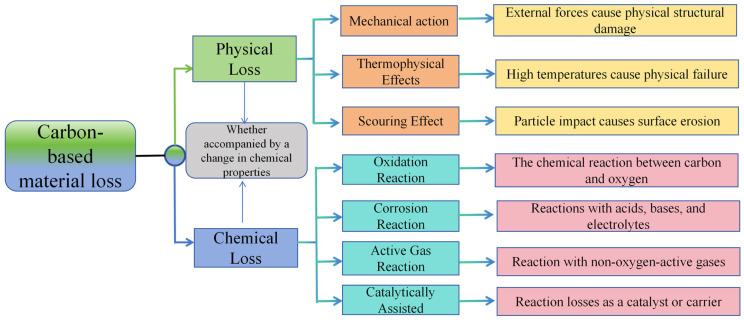
Classification of carbon material losses.

The loss of carbon-based materials can be divided into physical loss and chemical loss. The physical loss of carbon-based materials refers to the structural or morphological damage caused by external physical effects without chemical properties, resulting in performance degradation or mass loss. Physical loss only changes the physical state of the material; its chemical composition and basic properties remain unchanged, and the damaged part is still the original carbon-based component. Contrary to physical loss, chemical loss refers to the loss caused by the change in chemical composition due to the chemical reaction between carbon-based materials and substances in the surrounding environment. Chemical loss will destroy the chemical structure of the material and generate new substances, resulting in changes in the essential properties of the material (Summarized in Figure 5).

The core difference between the two is whether it is accompanied by changes in chemical properties: physical loss is the destruction of the physical form of the material and does not produce new substances. Chemical loss involves chemical reactions, forming new chemical substances, which are often irreversible and have a more fundamental impact on material properties. The main loss types and specific performances of different carbon-based materials will be different (as shown in the following Table 4).

In the treatment of pollutants, the irreversible deterioration of physical structure, chemical composition, and functional properties of carbon-based materials due to time, environment, or mechanical stress will bring multi-dimensional effects. The core effect is a significant decrease in pollutant removal efficiency. The removal ability of materials such as activated carbon and carbon nanotubes depends on the developed pore structure, huge specific surface area, and rich surface functional groups, and physical loss will destroy these structures. Abulikemu et al. [35] reported that after ball milling coal-based activated carbon (F400), the specific surface area decreased by 23–31%, and the micropore volume reduced by approximately 40%. The study of Xing et al. [36] showed that the BET surface area of the material was significantly reduced after mechanical stress or surface wear. In the nitrogen adsorption–desorption experiment of Guo et al. [37]. The surface area of Co9S8-CF composite material is 61.1312 m^2^/g, while the surface area of CF is only 4.7192 m^2^/g, which will lead to a significant increase in operating costs. In order to maintain the treatment effect, it is necessary to regenerate or replace the material more frequently. Regeneration consumes a lot of energy and chemicals, and replacement directly increases the purchase cost of new materials. More seriously, the risk of secondary pollution is increasing. The micron and nanometer carbon particles produced by physical loss may enter the environmental water body or air, which have potential ecological toxicity and are easy to become the carrier of other pollutants. Researchers have discovered that carbon nanoparticles can penetrate cell membranes and induce oxidative stress. In experiments with A549 cells, carbon nanotubes (CNTs) caused a 40% increase in reactive oxygen species (ROS) levels and a 30% rise in DNA damage rates [38]. Chemical loss will cause desorption or “leakage” of pollutants that are not firmly adsorbed or bound on the surface. The loss of materials containing special additives or catalytically active components may also release additives, bringing additional ecological toxicity risks [39].

### 3.2. Physical Loss Mechanism

**Figure 6:**
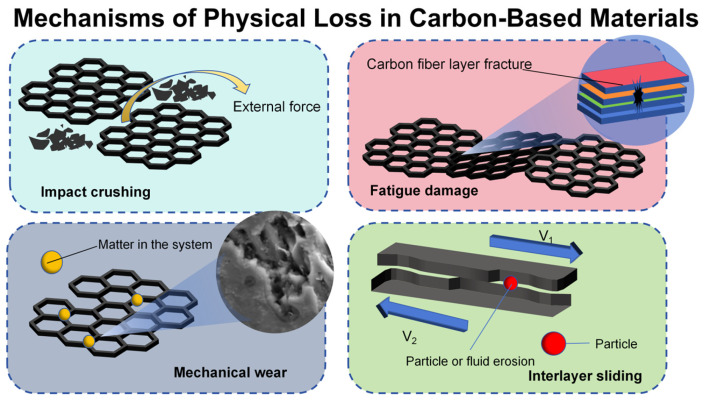
Physical loss mechanism diagram of carbon-based materials.

The core of the physical loss of carbon-based materials in the process of pollutant remediation is that the physical destructive force of the material exceeds its structural tolerance threshold, causing irreversible mass loss, pore network collapse and overall strength attenuation [40] is damage is not caused by chemical changes, but by the external kinetic energy directly acting on the material body, gradually weakening its pollutant retention capacity. It can be mainly divided into four types of mechanical wear, fatigue damage, impact crushing, and interlayer sliding loss (as shown in the Figure 6).

The connotation principle mechanism of different loss types is different, and the structural tolerance threshold can be calculated by different formulas (As shown in Table 5).

In terms of mechanical wear, when the solid-containing fluid flows through the surface of the carbon-based material, the suspended particles act as abrasives to continuously rub the surface of the material. The surface layer of the carbon skeleton gradually becomes thinner under repeated scraping, and the surface asperities are smoothed, resulting in the loss of active sites [44]. For porous activated carbon, this kind of wear will expand the pore entrance size, destroy the internal pore structure, and eventually cause the specific surface area to decrease. Fatigue damage is caused by frequent adsorption–regeneration cycles and pulse backwashing, which cause the material to bear alternating stress. Cannon et al. [45] found that thermal regeneration (800–900 °C) resulted in a 35% reduction in the micropore volume of activated carbon, leading to a 17–35% decrease in adsorption capacity for VOCs such as methyl isobutyl ketone. After more than 10 regeneration cycles, the particle breakage rate increased significantly, with mechanical strength declining by 20–30%.

At the micro level, stress is concentrated at grain boundaries and defects, inducing micro-crack initiation and gradual expansion. As the number of cycles increases, microcracks are connected to each other to form macroscopic cracks, which eventually lead to particle fracture or filter membrane perforation. This process has the characteristics of gradualness and concealment [46]. Impact crushing is the explosive concentration of local stress at the impact point when large particles in high-speed fluids or high-pressure water jets impact carbon materials with high kinetic energy [47]. When this stress exceeds the brittle fracture limit of the carbon material, it causes particle disintegration or overall structure crushing, which is particularly significant in the flow velocity mutation area. Dispersion loss mainly occurs in the field of nanoscale carbon materials. In strong shear fluids, such materials are prone to de-agglomeration. Because the micro-nano units combined by van der Waals force are separated by fluid kinetic energy, submicron fragments are formed to escape with the water flow [48]. His process not only causes the non-recyclable loss of active components, but also increases the turbidity of effluent. Interlayer slip lossFor multi-layer carbon matrix composites, under the action of alternating stress, the interlayer interface will produce shear slip. Repeated sliding will lead to the wear of the resin and the formation of wear debris, which will block the adjacent pores. At the same time, the debonding between the fiber and the matrix forms a micro-gap, which reduces the load transfer efficiency. The continuous expansion of the layered area will eventually lead to the overall warpage failure of the filter layer.

### 3.3. Chemical Loss Mechanism

The chemical loss of carbon-based materials refers to the process of mass loss, structural damage, or performance degradation due to irreversible chemical reactions under specific chemical environments. The essence of this loss is the reaction of carbon atoms or carbon skeletons with chemicals in the surrounding medium, which changes the chemical composition and microstructure of the material. The typical loss types can be divided into several categories in Table 6.

Among them, the loss of carbon-based materials due to oxidation, acidbase corrosion, and hydrolysis is the most common. In the field of pollutant treatment, the oxidation loss of carbon-based materials is a process involving complex chemical reactions and structural changes [49]. The carbon element in the carbon-based material undergoes an electron transfer reaction with the oxidant, destroying the carbon skeleton or the degradation of the surface functional groups. According to the type of oxidant, it can be divided into two mechanisms: free radical oxidation and direct electron transfer. In free radical oxidation, strong oxidizing substances such as ozone and hydrogen peroxide will produce hydroxyl radicals, attack unsaturated bonds or defect sites on the surface of carbon-based materials, trigger chain reactions, and gradually break the carbon skeleton into carbon dioxide and water [50]. Under the direct electron transfer mechanism, molecular oxygen or high-valence metal ions oxidize carbon to carbon monoxide or carbon dioxide by directly obtaining electrons from carbon. The two mechanisms are as follows:

Free radical oxidation mechanism (R is a carbon chain structure containing unsaturated bonds)

1. Hydroxyl radical generation:

O_3_(g) + H_2_O(l) → 2·OH(aq) + O_2_(g) (14)

2. Hydroxy radical attacks on carbon materials:

R-CH=CH-R′(s) + ··OH(aq) → R-CH(OH)-CH-R′(s)(15)

3. Chainwise oxidation and peroxyl radical generation:

R-CH(OH)-CH-R′(s) + O_2_(g) → R-CH(OH)-CH(O-O)-R′(s) (16)

R-CH(OH)-CH(O-O)-R′(s) + R″-CH-CH-R‴(s) → R-CH(OH)-CH(O-O-CH-R″-CH R‴)(s)(17)

4. Carbon skeleton fracture:

(18) $$R\text{-}\mathrm{CH}\left(\mathrm{OH}\right)\text{-}\mathrm{CH}(O\text{-}O\text{-}\mathrm{CH}\text{-}R^{\prime }\cdot )\left(s\right)\to R\text{-}\mathrm{CHO}\left(s\right)+\cdot \mathrm{CH}\left(\mathrm{OH}\right)\text{-}\mathrm{CH}\text{-}R^{\prime }(s)$$

Direct electron transfer mechanism (C is an amorphous carbon skeleton)

1. Oxidation process:

C(s) + 4OH^−^(aq) → CO_2_ + 2H_2_O + 4e^−^ (19)

2. Reduction process:

O_2_(g) + 2H_2_O(l) + 4e^−^ → 4OH^−^(aq) (20)

Total reaction:

(21) $$C\left(s\right)+O_{2}\left(g\right)\overset{Alkaline\;environment}{\to }{CO}_{2}(g)$$

Oxidation loss has many effects on pollutant treatment. From the structural point of view, it can lead to the collapse of the porous structure of carbon-based materials, the micropores of activated carbon expand into mesopores or macropores, the specific surface area is greatly reduced, and the adsorption capacity of small molecule pollutants is reduced. From the functional level, the active functional groups on the surface of the material are oxidized and lose the adsorption and catalytic ability of heavy metals or organic pollutants; at the same time, the gas and dissolved substances produced by oxidation may also cause secondary pollution, affecting the balance of the treatment system.

**Figure 7:**
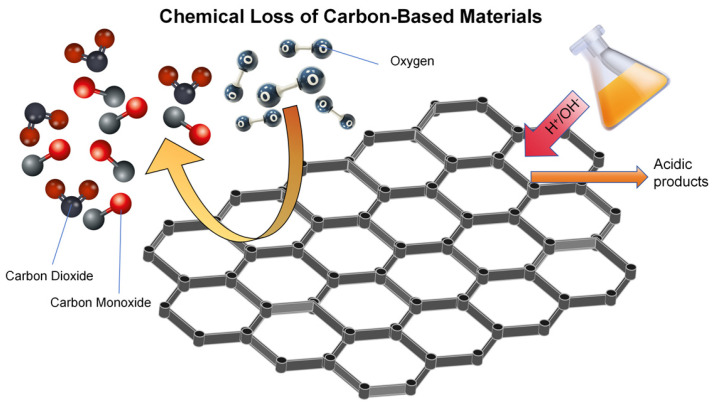
Scheme of chemical loss mechanism of carbon-based materials.

The acid/base corrosion loss of carbon-based materials in pollutant treatment is essentially structural damage or performance degradation caused by chemical reactions between materials and acid or alkali solutions. The mechanism can be analyzed from protonation and acid hydrolysis under acidic conditions, nucleophilic substitution, and saponification under alkaline conditions. In an acidic environment, H can attack the basic functional groups, such as amino and phenolic hydroxyl groups, on the surface of the material to make it protonated, weakening the adsorption capacity of pollutants [51]. Abonate impurities in carbon-based materials undergo a metathesis reaction with acid to form soluble salts and CO_2_, resulting in a loose structure. At the same time, acidic conditions can catalyze the hydrolysis reaction of easily hydrolyzed groups such as ester groups and amide groups, destroying the stability of molecular chains. In an alkaline environment, OH as a strong nucleophile attacks carbonyl-containing functional groups, triggering a nucleophilic substitution reaction, resulting in molecular chain breakage (R-COO-R′ OH^−^ → R-COO^−^ + R′-OH) [52]. The composites containing silicon and metal oxides can react with strong alkali to form soluble salts, resulting in skeleton dissolution and structural collapse (It can be simply illustrated as shown in Figure 7).

## 4. Strategy of Modification of Carbon-Based Materials

### 4.1. Necessity of Modification of Carbon-Based Materials

In the above research, it is not difficult to find that the physical and chemical loss of carbon-based materials directly affects their ability in catalytic adsorption, making it difficult for carbon-based materials to meet the actual needs in the corresponding core scenarios, which has become a key bottleneck restricting industrial applications. Therefore, the modification of carbon-based materials to reduce the loss has become one of the core areas in the field of carbon-based materials research.

In actual production applications, it is found that activated carbon is heated to about 430 °C by surface heat transfer with the high-temperature air provided by the outside in the analytical tower. During the negative pressure operation of the regeneration tower, the oxygen in the external air can penetrate the tower and oxidize the activated carbon, resulting in chemical loss [53]. Luo et al. [54] prepared cotton-based activated carbon fibers by the urea-enhanced low-temperature hydrothermal carbonization activation method. X-ray photoelectron spectroscopy and elemental analysis studies have shown that the form of nitrogen undergoes transformation and decomposition during high temperature, resulting in C-N separation. Thermogravimetric mass spectrometry characterization analysis found that potassium, carbon monoxide, and low-temperature hydrothermal-assisted KOH activation may fully react with the N-containing groups generated during the carbonization process to form NH_3_, resulting in more pore structures in carbon materials [55], effectively reducing the loss of carbon-based materials in practical production applications.

Excluding the impact of the loss of carbon-based materials, the nature of the carbon-based material itself also has limitations, and further modification is needed to improve its adsorption effect. From the perspective of performance optimization, the inherent properties of carbon-based materials make it difficult to meet the requirements of specific applications. The modification of activated carbon has improved the specific surface area and adsorption capacity of activated carbon. Huang Bangfu et al. [56] prepared nickel-loaded activated carbon by the nickel nitrate impregnation method. The simulated sintering flue gas desulfurization experiment showed that with the ordinary activated carbon at 30 °C, the desulfurization rate of more than 80% can be maintained for 19 min, and the sulfur capacity is 4.22 mg/g; meanwhile, with the modified activated carbon at 60 °C, the desulfurization rate of more than 80% can be maintained for 147 min, and the sulfur capacity is 54.18 mg/g. The nickel-loaded activated carbon modified by HNO_3_, at 60 °C, maintains a desulfurization efficiency of more than 80%, which can be maintained for 132 min, and the sulfur capacity is 62.21 mg/g, which intuitively reflects the significant difference in desulfurization performance before and after modification. It can effectively enhance the utilization rate of activated carbon in sintering flue gas desulfurization and denitrification.

From the perspective of application expansion, a single original carbon-based material is not enough to meet diverse needs. In the field of the metallurgical industry, efficient smelting requires higher thermal shock resistance and corrosion resistance of materials [57]. The original carbon-based refractory has obvious defects. After modification, carbon-based functional materials are applied to key parts such as the blast furnace and steelmaking furnace.

From the perspective of environment and sustainable development, modification is also an important way to improve the practicality and economy of carbon-based materials. The preparation process of many carbon-based materials may have problems of high energy consumption, high cost, or certain environmental impact on the environment. Modification can optimize the preparation process and improve the utilization efficiency of materials. At the same time, the modified carbon-based materials can treat environmental pollutants more efficiently, such as playing a greater role in catalytic degradation of organic wastewater and adsorption of heavy metal ions, helping environmental protection and green development.

It can be seen that the modification of carbon-based materials is an inevitable choice to cope with their performance limitations, meet the diversified application needs, promote technological progress in related fields, and achieve sustainable development. Through modification, it can not only explore the potential properties of carbon-based materials and expand their application range, but also provide strong material support for solving key problems in energy, environment, metallurgical industry, and other fields, which has significant scientific significance and practical application value.

### 4.2. Physical Modification

The modification methods of activated carbon can be divided into two categories: physical modification and chemical modification. This section will detail the physical modification (The specific classifications are shown in Table 7). Physical modification is intended to achieve pore structure optimization, surface impurity desorption, or morphology adjustment by using high temperature, gas activation, microwave, ultrasonic, mechanical force, and other physical effects without introducing new chemical groups. This process enhances the specific surface area, adsorption selectivity, or stability of activated carbon by etching, reconstructing pores, and increasing the degree of graphitization.

**Figure 8:**
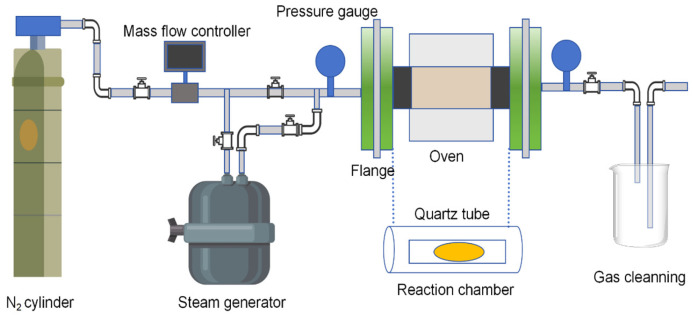
Scheme diagram of the activation device.

The core role of its physical modification is the following two points: The first is to make the tar and ash in the pores volatilize and activate the pores by means of high temperature and specific gas. The second is that in the process of activation, new pores are continuously generated, and the original pore wall is further etched so that the surface area of activated carbon per unit mass is greatly increased. Lan et al. [61] studied the effect of CO_2_ activation temperature on the structure and properties of blue coke-based activated carbon. The results showed that with the increase in activation temperature, the yield of activated carbon decreased, and the specific surface area and pore volume increased first and then decreased. When the activation temperature is 850 °C, the specific surface area of activated carbon reaches the maximum value of 592.26 m^2^/g, and the pore volume is 0.327 cm^3^/g. Guo et al. [62] studied the effect of CO_2_ activation on the pore structure of coconut shell activated carbon. It was found that increasing the activation temperature was beneficial to the formation of pores, thereby increasing the specific surface area, total pore volume, and micropore volume of activated carbon (As shown in Figure 8).

The physical modification of activated carbon has significant advantages in the metallurgical field and is highly suitable for the special needs of metallurgical production. By adjusting the pore structure, it can accurately match the demand for adsorption and recovery of precious metals in metallurgy, improve the enrichment efficiency of precious metals, and reduce the waste of resources. At the same time, the modified activated carbon has strong mechanical strength and thermal stability, and can withstand the high temperature and acid/base environment in the metallurgical process. It is not easy to damage and can be regenerated repeatedly, reducing the cost of consumables replacement. In addition, physical modification has no chemical reagent residue, which can avoid pollution to the purity of metallurgical raw materials and ensure the quality of metal products. It can also efficiently adsorb heavy metal ions in metallurgical wastewater and harmful components in waste gas, helping metallurgical enterprises to achieve environmental protection standards, taking into account production efficiency and environmental governance needs.

### 4.3. Chemical Modification

Chemical modification of activated carbon is a technology that changes the surface chemical composition, functional group type, and distribution of activated carbon through chemical reagents or chemical reactions (This can be summarized in Table 8). At the same time, the pore structure can be fine-tuned to optimize its adsorption, catalysis, ion exchange, and other properties. Compared with physical modification, it focuses more on enhancing the adsorption selectivity and capacity of substances by introducing active functional groups such as hydroxyl, carboxyl, and amino groups, or loading metal ions or oxides.

**Figure 9:**
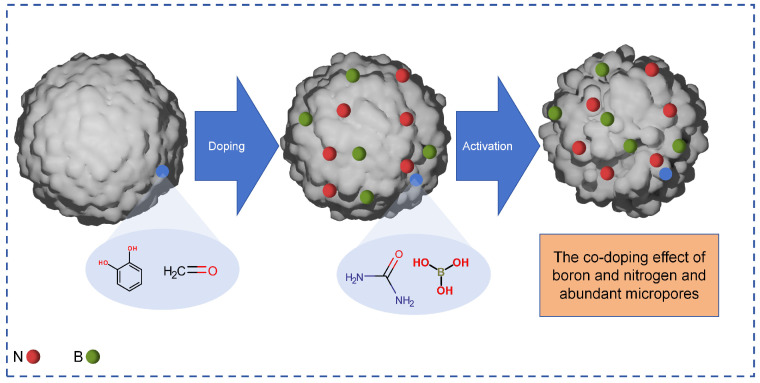
Schematic diagram of nitrogen co-doped porous carbon material (PRNB) transformation.

The core role of chemical modification of activated carbon lies in the following two points: The first is to react with the carbon element on the surface of activated carbon by means of chemical reagents, or to introduce specific functional groups such as hydroxyl, carboxyl, and amino groups by means of loading and grafting, and to remove inert impurities on the surface at the same time to achieve accurate regulation of surface chemical properties. Secondly, in the process of functional group modification or loading, some chemical effects can synchronously fine-tune the pore structure, and the functional group itself can enhance the adsorption force on the target substance, which greatly improves the adsorption selectivity and capacity of activated carbon for specific pollutants. Activated carbon materials not only serve as a medium for pollutant treatment themselves, but also function as highly efficient energy storage and conversion devices, thereby addressing environmental issues caused by fossil fuel combustion. Sun’s team [65] synthesized a porous carbon material named boron–nitrogen co-doped porous carbon (PRNB) through a two-step carbonization process using self-prepared phenolic resin. The PRNB material exhibits an exceptionally high specific capacitance of 330 F g^−1^ at 1 A g^−1^. Compared to other materials, it also exhibits outstanding rate capability (70%) and stability, with only 6% capacity loss after 10,000 cycles. Symmetrical electrodes based on PRNB achieved an energy density of up to 29.7 Wh kg^−1^ in neutral electrolytes (As shown in Figure 9).

Guan et al. [66] modified waste activated carbon with 10% sodium hydroxide, and the maximum saturated adsorption capacity of activated carbon to toluene reached 74.21 mg·g^−1^, which was higher than that of unmodified activated carbon. In the study, Lin [67] pointed out that activated carbon was prepared from sludge and areca straw, and FeCl_3_ was loaded by the impregnation method to obtain FeCl_3_-activated carbon (FAC). Fe^3+^ loading increased the specific surface area of FAC from 341.30 m^2^/g of AC to 442.15 m^2^/g, and the micropore volume increased by 28.7%. SEM showed that FeCl_3_ was evenly distributed on the pore surface. The saturated adsorption capacity of FAC for methylene blue was 341.30 mg/g (AC was 133.33 mg/g), and the adsorption efficiency was significantly improved in the range of pH 4–10. The Petrović team [68] successfully synthesized FeMg-PHC adsorbents from waste grape pomace using a two-step process combining hydrothermal carbonization with Fe/Mg metal salt doping and pyrolysis. These adsorbents demonstrated high efficiency in removing Pb^2+^ from aqueous solutions. Characterization analyses confirmed chemical adsorption and ion exchange as the primary mechanisms. Adsorption experiments revealed optimal removal efficiency at pH = 5, with the adsorption process following pseudo-second-order kinetics. The Sips model fitted a maximum adsorption capacity of 157.24 mg/g. This material offers a simple preparation method and excellent adsorption performance, demonstrating promising potential for lead-containing wastewater treatment.

## 5. Conclusions and Prospects

The existing carbon-based materials exhibit dual characteristics in pollutant treatment: on one hand, they can simultaneously remove multiple harmful substances from water, air, and soil at low cost, with activated carbon itself offering significant price advantages; on the other hand, constrained by traditional processes, carbon-based materials suffer from losses during application, and improper operation may generate byproducts like CO and CO_2_, leading to secondary pollution. Therefore, enhancing the comprehensive performance of carbon-based materials in pollutant treatment remains a key direction for current process optimization.

Modifying carbon materials serves as a vital pathway to enhance their performance, not only effectively boosting pollutant removal efficiency but also significantly improving their regenerative capacity. Carbon materials inherently possess unique physicochemical properties, exhibiting outstanding adsorption, catalytic, and reductive capabilities. Through various modification techniques, their reactivity can be further enhanced, accelerating the conversion rates of pollutants like sulfur and nitrogen. Modified carbon-based materials also reduce the formation of end-product byproducts, enhance microwave absorption capacity, and increase surface active sites. This comprehensively boosts pollutant adsorption rates, meeting the demands for low-cost, high-efficiency, highly stable, and environmentally friendly treatment.

Carbon-based materials retain vast development potential and room for in-depth exploration in pollutant treatment and modification research. Future efforts should focus on advancing modification and regeneration technologies toward greener, smarter approaches. Specifically, modification processes must gradually replace traditional strong acid/base methods by promoting low-energy, low-pollution techniques like plasma modification, ultrasonic-assisted activation, and bio-modification. This will achieve an optimal balance between efficiency and cost while accelerating industrial application. Simultaneously, systematic summarization of the strengths and limitations of the existing processes in practical applications is essential, with continuous optimization of technical routes based on industrial demands. Given the outstanding performance of supported non-metallic oxides and microwave irradiation technology in pollutant removal, it is necessary to further upgrade related process equipment to provide more effective technical support for pollution control in high-pollution sectors such as the steel industry.

## Figures and Tables

**Table 1:** Types of water pollution and its impact on the environment.

Pollution Type	Main Components and Sources	Environmental Impact
Heavy metal ion pollution	Pb, Cd, Hg, Cr, As, etc., source: industrial wastewater, mining, electronic waste, etc.	Disrupts soil structure and affects water quality
Organic pollutant pollution	Polycyclic aromatic hydrocarbons (PAHs), pesticides (DDT and organophosphorus), plasticizers (phthalates), microplastics, etc.; source: industrial wastewater, agricultural pesticide use, plastic waste, etc.	Difficult to degrade, prone to causing compound pollution.
Nitrogen and phosphorus nutrient pollution	Nutrient elements such as N and P; source: agricultural fertilizer loss, domestic sewage, industrial wastewater (such as food processing and detergent).	Water bodies are eutrophic, and water quality is deteriorating.

**Table 2:** The performance and principle of SO_2_, NO, and VOC hazards to the atmosphere [15,16,17].

Contaminant	Hazard Performance	Harm Principle
SO_2_	1. Forming sulfuric acid rain. 2. Participate in the formation of secondary particulate matter. 3. Reducing atmospheric visibility.	1. Oxidation in the atmosphere to form SO_3_, combined with water vapor to form H_2_SO_3_ aerosol or acid rain. 2. React with ammonia and metal ions in the atmosphere to produce secondary particles such as ammonium sulfate and iron sulfate, aggravating haze. 3. Particles scatter and absorb light to reduce atmospheric visibility.
NO_x_	1. Forming nitric acid rain. 2. Drive photochemical smog formation. 3. Participate in ozone pollution. 4. Promoting the formation of secondary particles.	1. NO_2_ reacts with water vapor to form HNO_3_ and NO, and HNO_3_ participates in the formation of acid rain. 2. Photochemical reaction occurs with VOCs under ultraviolet light irradiation, generating strong oxidizing pollutants such as O_3_, PAN, and forming photochemical smog. 3. As a key precursor of ozone formation, it promotes the increase in tropospheric O_3_ concentration.
VOCs	1. Participate in the generation of photochemical smog and ozone. 2. Promoting the formation of secondary organic aerosol (SOA). 3. Some VOCs have a greenhouse effect.	1. As the core precursor of photochemical reaction, it reacts with NO under light to continuously generate O_3_ and other oxidizing species. 2. Low volatile organic compounds are generated by oxidation reaction, and SOA is formed through condensation, adsorption, and other processes to increase the concentration of atmospheric particulate matter. 3. Some VOCs, such as methane and freon, can absorb infrared radiation and aggravate the greenhouse effect.

**Table 3:** Comparative properties of biochar, clay minerals, and zeolite composites [29,30,31].

Indicator	Biochar	Clay Minerals	Zeolite Composite Materials
Adsorption mechanism	Chemical complexation, ion exchange, and precipitation to prevent migration	Cation exchange, complexation	Ion exchange and adsorption binding
Adsorption capacity	Affected by functional group density and preparation conditions	Higher, but easily constrained by acidic or alkaline environments	Highest capacity, but relatively high cost
Environmental Adaptability	With changes in pyrolysis conditions and pH value	Performance declines under acidic or high-salt conditions.	Structures may become unstable in acidic and strongly saline environments.
Preparation and Cost	The preparation technology is mature and low-cost.	Abundant natural resources, low costs	Complex manufacturing process, high cost
Application Prospects	High repair efficiency, with modified technology to adapt to varying pollution conditions	Suitable for rapid repair under neutral conditions	It performs exceptionally well in water treatment and can be extended to specific complex systems.

**Table 4:** Loss types and specific performance of carbon-based materials.

Material Type	Main Loss Modes	Embodiment
carbon fiber composite	mechanical wear	The fiber and resin interface debonding and fiber fracture result in a decrease in material strength.
	environmental erosion	Humidity, salt fog, and so on lead to resin degradation and fiber oxidation corrosion.
	Fatigue damage	Micro-cracks are generated and propagated under cyclic loading, causing structural failure.
Carbon nanotube materials	Dispersion loss	The agglomeration of nanotubes leads to a decrease in electrical/thermal conductivity, and an improper dispersion process leads to performance degradation.
	Chemical oxidation	Strong oxidant destroys the structure of carbon nanotubes and affect the electrical properties.
	High temperature degradation	When the temperature exceeds the tolerance temperature, the carbon tube structure collapses or transforms into other carbon forms.
Graphene materials	Layer increase/defect generation	In the preparation process, the number of stacked layers increases, or mechanical stripping produces lattice defects, resulting in performance degradation.
	Physical adsorption saturation	As an adsorption material, the surface functional groups are occupied by pollutants and lose their adsorption capacity.
	Interlayer sliding loss	Multilayer graphene has interlayer dislocations under the action of shear force, which affects the overall mechanical and electrical properties.
activated charcoal	pore plugging	During the adsorption process, micropores are filled with macromolecular contaminants, resulting in a decrease in adsorption capacity.
	Mechanical crushing	In a high flow rate fluid or vibration environment, granular activated carbon is worn and broken, which reduces the efficiency of use.
	Regenerative failure	In the process of high temperature or chemical regeneration, the structure of activated carbon collapses, and the adsorption performance cannot be restored.
Diamond (carbon material)	heat injury	At high temperatures, it reacts with oxygen to form CO_2_, resulting in a decrease in hardness and optical properties.
	impact crusher	Brittle fracture leads to the collapse of diamond particles and the loss of cutting ability.
	chemical corrosion	In a strong acid/base environment, the carbon atoms on the surface of the diamond are eroded, and the crystal structure is destroyed.

**Table 5:** Physical loss mechanism and its principle [41,42,43].

Loss Type	Mechanism of Action	Related Formulas and Principles
Mechanical wear	Friction causes surface particles to peel off, deform, or break.	Archers equation: Q = kFL/H Adhesive wear: shear separation after plastic deformation of asperities
Fatigue damage	Microcrack propagation induced by cyclic loading	Paris law: da/dN = C(ΔK)^m
Impact crushing	Instantaneous impact induced stress super strength limit	Stress wave theory: When the amplitude of the stress wave exceeds the dynamic strength of the material, the fracture occurs.
Interlayer sliding loss	The shear force makes the multi-layer materials dislocated between layers.	Shear force timeout sliding.

**Table 6:** Chemical loss mechanism and performance impact.

Depletion Type	Mechanism of Action	Typical Conditions	The Impact of Performance	Common Carbon-Based Materials
Oxidation loss	Carbon materials react with oxygen/oxidant to produce CO_2_, CO, or carbon oxides.	High temperature, strong oxidant, combustion environment	Mass loss, strength decrease, surface roughness increase, and conductivity decrease	Carbon fiber, graphene, diamond, carbon nanotubes
Acid/base corrosion	Carbon materials react with strong acids/alkalis (such as oxidation and proton exchange).	Strong acid, strong alkali	Structural fragmentation, introduction of functional groups, and increased porosity	Activated carbon, carbon felt, carbon composite materials
Hydrolysis loss	Water molecules react with functional groups (such as ester groups and hydroxyl groups) on the surface of carbon materials.	High humidity environment, high temperature water/steam, acidic/alkaline aqueous solution	Interface debonding (composite material), polymer chain fracture, and mechanical properties degradation	Carbon-polymer composites
Photochemical degradation	Light energy (ultraviolet/visible light) triggers electronic excitation or free radical reaction of carbon materials.	UV irradiation (e.g., outdoor environment), presence of photosensitizer	Structural defects (such as vacancies and broken bonds), increased oxidation, and changes in optical properties	Graphene film, carbon-based optoelectronic devices
Electrochemical corrosion	The electrochemical reaction of carbon materials in the electrolyte solution occurs.	Electrochemical environment	The mass loss of electrode materials and the reduction in electrochemically active sites	Carbon electrode
Biodegradation	Microbial decomposition of carbon-based materials	Wet soil, in vivo environment, microbial-rich medium	The molecular chain breaks, and the mass gradually disappears	Bio-based carbon materials
Irradiation chemical damage	High-energy rays cause carbon bond breakage or cross-linking	Nuclear radiation environment, particle accelerator, space radiation	Free radical formation, structural disorder, and mechanical properties degradation	Nuclear graphite, aerospace carbon materials

**Table 7:** Physical modification methods and advantages [58,59,60].

Modification Method	Modification Principle	Advantages
High-temperature heat treatment	The activated carbon was heated under the protection of inert gas to promote the decomposition of unstable groups in the activated carbon, adjust the pore structure, reduce the surface heteroatoms, and improve the degree of graphitization.	It can significantly improve the thermal stability and chemical stability of activated carbon, expand the pore size and optimize the pore distribution, enhance the adsorption capacity of macromolecular substances, and there is no chemical pollution in the modification process.
Steam activation method	Using water vapor to react with carbon atoms on the surface of activated carbon at high temperature, new pores are formed on the etched surface, or the original pores are expanded to increase the specific surface area.	It can effectively increase the specific surface area and total pore volume, generate abundant micropores and mesopores, and have good adsorption effects on polar and non-polar substances. The process is mature and easy to scale.
CO_2_ activation method	At high temperature, CO_2_ reacts with carbon atoms in activated carbon, expands pores by selective etching, and regulates pore size and distribution.	It can precisely control the pore structure, generate more uniform micropores and mesopores, and has strong adsorption selectivity and excellent adsorption performance for non-polar substances.
Microwave modification method	The thermal effect of microwave is used to rapidly heat up the interior of activated carbon, causing a local high temperature to lead to pore structure reconstruction and promoting surface impurity desorption.	The heating speed is fast and uniform, which can shorten the modification time and avoid the thermal hysteresis of traditional heating. The adsorption rate of activated carbon after modification is significantly improved.

**Table 8:** Chemical modification methods and advantages [63,64].

Classification	Core Principle	Main Advantages
Oxidation modification	Oxygen-containing functional groups such as hydroxyl (-OH), carboxyl (-COOH), and carbonyl (C=O) were introduced by the reaction of oxidants (such as HNO_3_, H_2_O_2_, and O_3_) with carbon on the surface of activated carbon, and pores can be etched at the same time.	It significantly improves the adsorption capacity of polar substances (such as heavy metal ions and polar organic matter). The operation is relatively simple, and the introduction efficiency of functional groups is high.
Reduction modification	Some oxygen-containing groups were removed by the reaction of the reducing agent with oxygen-containing functional groups on the surface of activated carbon, or reducing functional groups, such as amino (-NH_2_), were introduced to adjust the surface charge properties.	Enhance the adsorption and reduction ability of oxidizing pollutants (such as Cr^6+^ and NO_3_^−^), and improve the adsorption selectivity of non-polar substances.
Load modification	Metal ions or metal oxides were loaded on the pore and surface of activated carbon by impregnation and precipitation, and the adsorption was enhanced by the coordination of metal ions or the catalysis of metal oxides.	It has both adsorption and catalytic properties, and the adsorption capacity and degradation efficiency of specific pollutants (such as VOCs and dyes) are greatly improved, and the selectivity is strong.

## Data Availability

Data sharing is not applicable.
